# Napping on the night shift and its impact on blood pressure and heart rate variability among emergency medical services workers: study protocol for a randomized crossover trial

**DOI:** 10.1186/s13063-021-05161-4

**Published:** 2021-03-16

**Authors:** P. Daniel Patterson, Leonard S. Weiss, Matthew D. Weaver, David D. Salcido, Samantha E. Opitz, Tiffany S. Okerman, Tanner T. Smida, Sarah E. Martin, Francis X. Guyette, Christian Martin-Gill, Clifton W. Callaway

**Affiliations:** 1 Department of Emergency Medicine, University of Pittsburgh, School of Medicine, 3600 Forbes Ave., Iroquois Building, Suite 400A, Pittsburgh, PA 15261 USA; 2 Division of Community Health Services, Emergency Medicine Program, University of Pittsburgh, School of Health and Rehabilitation Sciences, Pittsburgh, PA 15261 USA; 3 Division of Sleep and Circadian Disorders, Brigham and Women’s Hospital, Boston, MA 02115 USA; 4 Harvard Medical School, Division of Sleep Medicine, Boston, MA 02115 USA

**Keywords:** Crossover trial, Napping, Shift work

## Abstract

**Background:**

There is an emerging body of evidence that links exposure to shift work to cardiovascular disease (CVD). The risk of coronary events, such as myocardial infarction, is greater among night shift workers compared to day workers. There is reason to believe that repeated exposure to shift work, especially night shift work, creates alterations in normal circadian patterns of blood pressure (BP) and heart rate variability (HRV) and that these alterations contribute to increased risk of CVD. Recent data suggest that allowing shift workers to nap during night shifts may help to normalize BP and HRV patterns and, over time, reduce the risk of CVD. The risk of CVD related to shift work is elevated for emergency medical services (EMS) shift workers due in part to long-duration shifts, frequent use of night shifts, and a high prevalence of multiple jobs.

**Methods:**

We will use a randomized crossover trial study design with three study conditions. The targeted population is comprised of EMS clinician shift workers, and our goal enrollment is 35 total participants with an estimated 10 of the 35 enrolled not completing the study protocol or classified as lost to attrition. All three conditions will involve continuous monitoring over 72 h and will begin with a 36-h at-home period, followed by 24 total hours in the lab (including a 12-h simulated night shift), ending with 12 h at home. The key difference between the three conditions is the intra-shift nap. Condition 1 will involve a simulated 12-h night shift with total sleep deprivation. Condition 2 will involve a simulated 12-h night shift and a 30-min nap opportunity. Condition 3 will involve a simulated 12-h night shift with a 2-h nap opportunity. Our primary outcomes of interest include blunted BP dipping and reduced HRV as measured by the standard deviation of the inter-beat intervals of normal sinus beats. Non-dipping status will be defined as sleep hours BP dip of less than 10%.

**Discussion:**

Our study will address two indicators of cardiovascular health and determine if shorter or longer duration naps during night shifts have a clinically meaningful impact.

**Trial registration:**

ClinicalTrials.gov NCT04469803. Registered on 9 July 2020

**Supplementary Information:**

The online version contains supplementary material available at 10.1186/s13063-021-05161-4.

## Administrative information

**Table:** 

Title {1}	Napping on the night shift and its impact on blood pressure and heart rate variability among Emergency Medical Services workers: Study protocol for a randomized crossover trial
Trial registration {2a} and {2b}.	Our protocol has been registered with clinicaltrials.gov (registration number: NCT04469803; public release date: 9 July 2020) {2a} and {2b}. All subjects are required to provide written informed consent.
Protocol version {3}	Protocol version 1 as of February 2021 {3}.
Funding {4}	The ZOLL Foundation has provided funding for this study {4}. The ZOLL Foundation has no role in the study design, data collection, management, analysis, interpretation of results, writing of study findings, or decision on publication {5c}. The ZOLL Foundation can be reached at foundation@zollfoundation.org and by visiting the foundation website: www.zollfoundation.org {5b}.
Author details {5a}	P. Daniel Patterson, PhD, NRP^1,2^ Leonard S. Weiss, MD^1^ Matthew D. Weaver, PhD^3,4^ David D. Salcido, PhD, MPH^1^ Samantha E. Opitz, MS Tiffany S. Okerman, NRP^1,2^ Tanner T. Smida, EMT^1^ Sarah E. Martin, MPH^1^ Francis X. Guyette, MD, MPH^1^ Christian Martin-Gill, MD, MPH^1^ Clifton W. Callaway, MD, PhD^1^ 1: University of Pittsburgh, School of Medicine, Department of Emergency Medicine, Pittsburgh, PA 15261. 2: University of Pittsburgh, School of Health and Rehabilitation Sciences, Division of Community Health Services, Emergency Medicine Program, Pittsburgh, PA 15261. 3: Brigham and Women’s Hospital, Division of Sleep and Circadian Disorders, Boston, MA 02115. 4: Harvard Medical School, Division of Sleep Medicine, Boston, MA 02115.
Name and contact information for the trial sponsor {5b}	The ZOLL Foundation can be reached at foundation@zollfoundation.org and by visiting the foundation website: www.zollfoundation.org {5b}.
Role of sponsor {5c}	The ZOLL Foundation has no role in the study design, data collection, management, analysis, interpretation of results, writing of study findings, or decision on publication {5c}.

## Introduction

### Background and rationale {6a} {6b}

There is an emerging body of evidence that links exposure to shift work to cardiovascular disease (CVD) [1]. The incidence of hypertension (HTN) is higher among shift workers compared to persons who work traditional daylight schedules [2, 3]. Shift workers have a 23% higher risk of myocardial infarction and a 5% higher risk of stroke relative to day workers [4, 5]. In addition, the risk of coronary events (e.g., myocardial infarction, coronary mortality, hospital admission due to coronary artery disease) is greater among night shift workers compared to day workers (risk ratio 1.41, 95%CI 1.13, 1.76) [5]. There is reason to believe that repeated exposure to shift work creates alterations in the normal circadian patterns of blood pressure (BP) and heart rate variability (HRV) and that these alterations contribute to increased risk of CVD over time [6, 7]. There is also reason to believe that allowing shift workers to nap on duty (especially during night shift work) may help to normalize BP and HRV patterns and over time reduce the risk of CVD due in this population [8, 9].

Shift work refers to work schedules outside the traditional daylight schedule (e.g., 9 am to 5 pm) [10]. Paramedics and emergency medical technicians (EMTs) are shift workers within the United States Emergency Medical Services (EMS) system, which comprised more than one million certified/licensed personnel and approximately 20,000 EMS agencies nationwide [11]. The clinician shift workers who work in EMS are often deployed in crews of two and frequently work night shifts, evening shifts, long-duration shifts (e.g., 24 h and 48 h), and rotating or irregular shift schedules [12]. Shift work contributes to fatigue, excessive daytime sleepiness, and problems with obtaining adequate sleep [13, 14]. Previous research shows that three quarters of EMS shift workers report occupational fatigue, greater than half report poor sleep quality, half report inadequate recovery between scheduled shifts, and half report sleeping less than 6 h per night [15–19].

Shift work forces prolonged wakefulness and disrupts a number of biological, chemical/hormonal, and physiologic mechanisms that are closely tied to the night/day, sleep/wake cycle (circadian rhythms) [20, 21]. The normal circadian pattern of BP is characterized by elevations during daylight hours and wakefulness, followed by a decrease of 10–20% during nighttime hours and sleep [9, 22]. This decrease in BP during sleep and nighttime hours is referred to as the “dip” [9, 22]. Blunting of the BP dip occurs when BP fails to drop by 10% during nighttime hours or during sleep [9, 22]. In select populations, blunted BP dipping during sleep and nighttime hours has been linked to select demographic and physiologic factors, elevated levels of stress, stroke, target organ damage, left ventricular hypertrophy, progressive renal damage, and cardiovascular mortality [23–31]. Previous research of night shift workers (e.g., physicians and nurses) shows that nighttime BP is often elevated compared to daytime values [32, 33], and in some cases, the BP dip is blunted during shift work and during recovery immediately after shift work [34]. Our recent study of 56 EMS clinicians shows that a large proportion of those who work night shifts experience blunted BP dipping during shift work [35].

Previous research also shows an association between shift work and unhealthy HRV patterns [36–39]. Healthy HRV may be characterized by oscillations in the heart rate and beat-to-beat fluctuations that are more variable over a 24-h period [40–42]. Greater variability in patterns of HRV signifies balance between sympathetic and parasympathetic activity and is often associated with better health, better performance, and increased ability to respond to, and cope with stress [41]. Decreased variability, or reduced HRV, may be a marker of increased sympathetic activity and reduced parasympathetic activity. Prolonged periods of reduced HRV have been linked to acute cardiac events and all-cause mortality [43, 44]. One study of 14 EMTs revealed reduced levels of HRV during periods of sleep that occurred during shift work versus during sleep on non-workdays [39]. A study of nine paramedics in Japan showed an abnormal HRV pattern during the workday compared to the non-workday [45]. A separate study of 14 male firefighter-paramedics in Finland reported decreased variability in heart rate during a 24-h shift followed by normalization to healthier levels after 3 days of rest [36]. Together, these studies suggest that shift work may have a negative impact on the normal circadian pattern of HRV among EMS shift workers.

Shift work is essential for many occupations, including public safety workers such as police, fire, and EMS, which provide critical services that must be available all hours, day and night [46]. Many EMS employers would likely object to policies that restrict shift duration or timing as a means to guard against the impact of shift work on cardiovascular health [47]. Employers are more likely amenable to strategies that allow for shift work to continue and simultaneously reduce its impact on select outcomes. One such strategy is napping during shift work. Napping has been described as “sleep periods at least 50% shorter than an individual’s average nocturnal sleep length” [48, 49]. A recent systematic review and meta-analysis shows that napping during night shifts has a positive impact on shift worker’s subjective ratings of sleepiness and fatigue [50]. Evidence-based guidelines for fatigue risk management in EMS promote napping during shift work, especially during night shifts [51]. These data and guidance support intra-shift napping as it pertains to worker safety [51]. However, previous research devoted to the health benefits of intra-shift napping is limited. Employers face a great deal of uncertainty when weighing the risks versus benefits of intra-shift napping, and uncertainty regarding implementation.

### Objectives {7}

In this paper, we present the study protocol for an experimental study of EMS shift workers using intra-shift napping during simulated night shifts. We will use a randomized crossover study design comprised of two intra-shift napping conditions and one no-nap comparison condition. With this design, we first aim to determine the impact of different durations of napping during night shifts on BP dipping and HRV during sleep and during nighttime hours. Second, we aim to determine if longer duration naps are associated with improved recovery or a more rapid normalization of BP and HRV patterns post-shift work. We hypothesize higher odds of blunted BP dipping during the no-nap condition versus the napping conditions. We hypothesize that when compared to the napping conditions, select HRV measures taken during the no-nap condition (i.e., the standard deviation of the inter-beat intervals of normal sinus beats, SDNN) will be reduced. We hypothesize that BP and HRV patterns observed immediately following the simulated night shift for both napping conditions will normalize sooner than observed for the no-nap condition. Finally, we hypothesize that for both BP and HRV, the greatest benefit will be observed in the longer duration nap of 2 h versus the shorter 30-min nap condition.

### Trial design {8}

**Fig. 1:**
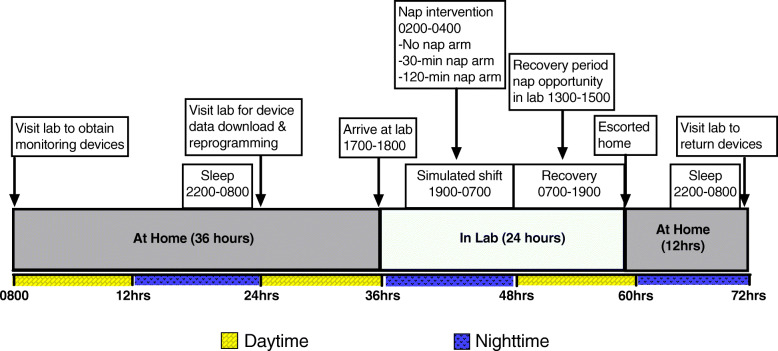
Study protocol timeline

**Fig. 2:**
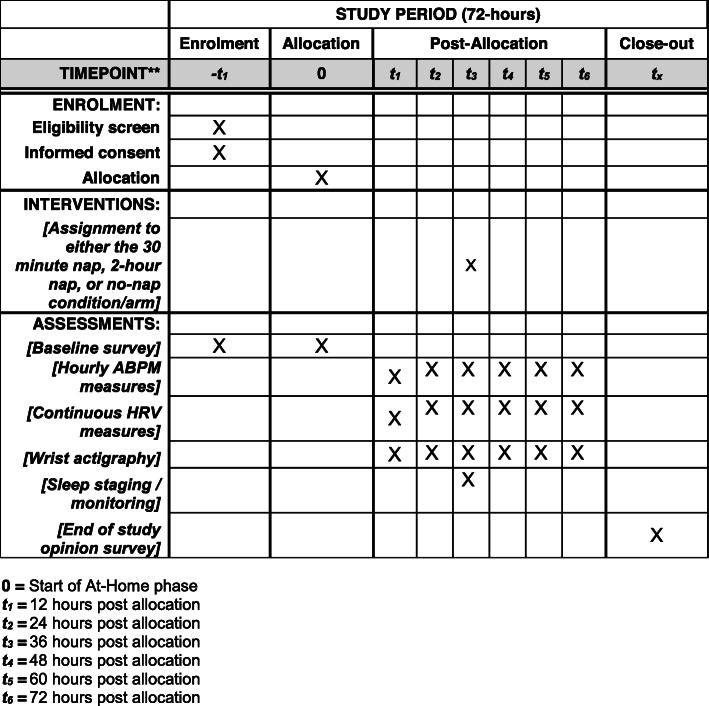
Schedule of enrolment, interventions, and assessments. 0 = start of at-home phase. *t*_*1*_ = 12 h post allocation. *t*_*2*_ = 24 h post allocation. *t*_*3*_ = 36 h post allocation. *t*_*4*_ = 48 h post allocation. *t*_*5*_ = 60 h post allocation. *t*_*6*_ = 72 h post allocation

We will use a randomized crossover trial study design with three study arms/conditions. All three conditions will involve continuous monitoring over 72 h and will begin with a 36-h at-home period, followed by 24 total hours in the lab (including a 12-h simulated night shift), and ending with 12 h at home. The key difference between the three conditions is the presence or absence of an intra-shift nap and the duration of that nap. Condition 1 will involve a simulated 12-h night shift with no nap: total sleep deprivation. Condition 2 will comprise another simulated 12-h night shift and a 30-min nap opportunity. Condition 3 will involve a simulated 12-h night shift and a 2-h nap opportunity. There will be a mandatory 1–3 week washout period between conditions. The order in which participants complete the study conditions will be balanced by a William’s square design. In the paragraphs below, we outline the details of our experimental study in accordance with the CONSORT/SPIRIT statement. Figure 1 illustrates the flow chart of our trial, and Fig. 2 illustrates the SPIRIT template for enrolment, intervention, and assessment. The University of Pittsburgh Institutional Review Board (IRB) approved this study {24}, and our protocol has been registered with ClinicalTrials.gov ({2a} registration number: NCT04469803; public release date: 9 July 2020). All subjects are required to provide written informed consent (see Additional file 1, which reports on all elements of the SPIRIT checklist). All elements of the World Health Organization Trial Registration Data Set can be found in this article, supplemental file, and the ClinicalTrials.gov registration.

## Methods: participants, interventions, and outcomes

### Study setting {9}

The study setting is inclusive of Western Pennsylvania in the USA. This area is home to more than 300 EMS agencies and more than 9000 certified EMS clinicians [52]. All laboratory-based procedures will occur on the University of Pittsburgh campus in the Department of Emergency Medicine’s Applied Physiology Laboratory.

In response to COVID-19, we will implement additional screening and in-laboratory procedures. These include the following: (A) during participant screening, we will give preference to eligible participants who have received at least one dose of the COVID-19 vaccine prior to starting the study. All non-vaccinated, yet otherwise eligible participants will be placed on a wait-list or be allowed to start the study after all vaccinated participants have completed the study protocol. (B) Prior to entering the laboratory, eligible participants will undergo temperature screening and questioning about recent illness, which will include any signs or symptoms of COVID-19 (e.g., fever or chills, cough, shortness of breath, body fatigue, muscle or body aches, headache, new loss of taste or smell, sore throat, congestion or runny nose, nausea, vomiting, or diarrhea). Participants who respond yes to one or more questions and/or a non-contact forehead temperature reading of > 100.3 °F (37.9 °C) will involve a consult with a licensed emergency medicine physician involved with the study protocol. (C) Masks must be worn by study staff and by study participants at all times when inside the laboratory. Participants may remove their mask while sleeping/napping or when not in the laboratory (e.g., at home). (D) We will limit the number of times and total amount of time that study staff are within 6-ft of study by instructing, demonstrating, and monitoring from a safe distance (> 6 ft) while participants apply select non-invasive measurement devices (e.g., Holter monitors, ambulatory blood pressure monitoring devices, and wrist actigraphy devices). Study staff will quickly validate placement following participant self-application. (E) Study staff will maintain a safe distance from study participants (> 6 ft) for the duration of the laboratory-based component of the study, except when absolutely necessary.

### Eligibility criteria {10}

Inclusion criteria include the following: (A) a commitment to 72 h of continuous monitoring on three separate occasions; (B) a commitment to complete a laboratory-based simulated 12-h night shift during each 72-h period; (C) a commitment to going without sleep for at least one of the simulated night shifts; (D) a commitment to wearing multiple monitoring devices (i.e., Holter monitors, blood pressure monitors) during each of the 72 h of monitoring; (E) a commitment to abstain from exercise, caffeine, alcohol, and nicotine during the 72-h study interval and to follow a structured sleep and food consumption protocol for 72 consecutive hours during each visit; (F) confirmation of current licensure/certification as an EMS clinician (i.e., EMT, paramedic, flight nurse, or other first responder); (G) confirmation of current work as a front-line clinician (with full-time administrators excluded); (H) lack of known medical conditions that may impact findings (i.e., a sleep disorder, hypertension, CVD, history of myocardial infarction or stroke, kidney disease, liver disease, adrenal disease, thyroid disease, rheumatologic disease, hematologic disease, cancer, or organ transplantation); (I) not known to be pregnant; (J) confirmation of no physical conditions that may prevent wearing of multiple monitoring devices; and (K) age 18 years or older.

### Who will take informed consent {26a}

All who are interested in participation will use email or telephone to initiate contact with the study team and schedule a time for screening and consenting. We will determine eligibility with a standardized screening form. A qualified member of the study team will be responsible for obtaining written informed consent (see Additional file 2 for a copy of consent form {32}). Randomization and scheduling will occur immediately thereafter for those determined eligible.

### Additional consent provisions for collection and use of participant data and biological specimens {26b}

This study protocol does not include the collection of biological specimens. The addition of ancillary studies would involve additional approval from the University of Pittsburgh IRB and additional consent from study participants.

## Interventions

### Explanation for the choice of comparators {6b}

There is reason to believe that if given the opportunity to nap during shift work, EMS shift workers may experience a benefit to their cardiovascular health. Both BP and HRV are tightly tied to timing, duration, and depth of sleep [9, 53]. Slow-wave sleep or deep sleep will often occur within 30 to 60 min after initiating sleep and is associated with a significant drop in BP [9]. Some research shows that slow-wave sleep can occur with a nap duration of < 30 min [8]. During this time, dynamic changes will arise in HRV with an increase in parasympathetic activity and a decrease in sympathetic activity [9, 53]. Given these data, a healthy (normal) drop in BP of 10–20% and normal circadian changes in HRV similar to non-workdays may be achievable during intra-shift naps, especially when utilized during night shifts. What is not yet clear is do EMS shift workers achieve a healthy dip in BP and a normal circadian change in HRV during shorter as well as longer duration naps?

### Intervention description {11a}

Our intervention of interest is intra-shift napping. All subjects in our study protocol will participate in two intervention nap conditions and one no-nap comparison condition. All nap opportunities will be available between 0200 and 0400 h during the in-laboratory phase and simulated night shift. We chose this time period given that (A) EMS call volume (workload) is often low or limited at this time, and many who are allowed to nap on duty may use this time period for sleep, and (B) this window of time coincides with a known time of high homeostatic sleep pressure and minimal circadian alerting signal [21]. Protocol conditions 2 and 3 will involve scheduled intra-shift nap opportunities of 30 min and 2 h, respectively. Our choice of 30 min and 2 h for the nap opportunities was based on the following: (A) previous research has suggested that short duration naps are optimal during night shift work for sustained or improved performance and avoidance of sleep inertia (that groggy feeling experienced upon waking) [8]; (B) shorter duration naps are attractive in public safety because EMS work is unpredictable and when called upon, an EMS worker must react quickly and make medical decisions to care for the acutely ill and injured; (C) many EMS employers who may not endorse sleeping while on duty might be amenable to short versus longer opportunities for intra-shift napping [51]; and (D) blood pressure and HRV are tightly tied to sleep and depth of sleep [9]. As described previously, normal dips in BP and parasympathetic-driven changes in HRV often occur when entering deeper, slow-wave sleep, which can occur 30 min or longer after beginning sleep [9, 53]. Therefore, the longer 2-h nap condition may be superior in terms of a cardiovascular-focused health benefit than would the shorter 30-min nap condition.

Following the initial 36 h at home, the laboratory phase will begin with participants arriving at the lab between 1700 and 1800 h. The simulated night shift will begin at 1900 and end at 0700 (12 total hours) the next day. Participants will follow study intervention procedures based on the order in which they were randomized (i.e., condition 1 = no nap, condition 2 = brief nap opportunity of 30 min, or condition 3 = longer nap opportunity of 2 h). We have designed the simulated night shift to include activities that are common during real work conditions. In most EMS operations, the dispatch (or call) volume is often reduced during nighttime hours. Night shifts for EMS shift workers often involve long periods of downtime with limited and unpredictable patient-related activity. During downtime, EMS shift workers read, watch television, use the Internet, or complete continuing education, or sleep if permitted. For purposes of this study, we will allow participants to engage in these activities with the exception of sleeping. Participants will sleep only when approved to do so for the two napping conditions. Our simulated night shift will involve four diverse patient encounters introduced at random times throughout the simulated night shift (minus the set-aside times for the napping conditions). We will separate these encounters throughout the shift so that no two occur back to back within a short time period. We will also introduce these mock patient encounters at similar time intervals for all study participants so that the workload does not differ in a meaningful way. Four patient encounters during nighttime hours is a moderate level of workload. The four mock patient encounters will include a low acuity patient encounter (i.e., a lift assist), a high acuity patient encounter (i.e., a cardiac arrest), and two moderate acuity patient encounters (i.e., a traumatic injury and a patient with a medical emergency). Study staff will administer these mock patient encounters with simulated “alarms” sounding as if the participant was at work. We will allow for the movement of the participant (and mock patient) within the laboratory space and the office space where the laboratory is located. Patient treatment (e.g., cardiopulmonary resuscitation) will be performed on a low-fidelity manikin. Study staff will use standardized checklists to document the actions of study participants in accordance with state- and national-level protocols for EMS clinical care. The simulated patient encounters will not involve operating an ambulance or driving simulator.

For conditions 2 and 3, the nap opportunities will be introduced between 0200 and 0400. In addition to wearing wrist actigraphy for sleep/wake monitoring, all participants will wear the portable Zmachine® Synergy machine provided by the General Sleep Corporation (Cleveland, OH). The Zmachine® Synergy is a non-invasive sleep staging device that produces numerous measures to quantify and differentiate light sleep (stages 1 and 2) from deeper sleep (stages 3 and 4) from rapid eye movement (REM) sleep. The Zmachine® Synergy will be used only when the participant is engaging in his/her sleep opportunity from 0200 to 0400 and during the recovery period of sleep. With these data, we will explore the association between stages of sleep (depth of sleep) and changes in BP and HRV during intra-shift napping/sleep.

At the end of the simulated 12-h night shift, participants will then enter a recovery period. This period entails participants remaining in the laboratory and maintaining wakefulness from 0700 to 1300 h. At 1300, we will provide participants with a 2-h recovery nap opportunity from 1300 to 1500. At 1900 h, we will escort participants to their homes, via paid transportation, for the final phase of the study arm. Participants will continue to be monitored with ABPM, HRV Holter monitoring, and actigraphy for another 12 h. During this period, participants will be asked to sleep, recover, and not engage in scheduled or unscheduled shift work, and avoid consumption of caffeine or alcohol and avoid nicotine. At 0800 h the following day, participants will return to the lab and complete study-related measurements and return study equipment. Throughout each 72-h period, participants will be instructed to follow a scheduled dietary plan. We will use a mandatory 1–3 week washout period between each of the three study conditions. Confidentiality of study participation will be maintained by storing all study-related forms in locked filing cabinets in the locked office of study investigators, located on the University of Pittsburgh campus. All data will be examined for accuracy (quality) and abstracted into electronic form in a University of Pittsburgh maintained version of REDCap (Research Electronic Data Capture) [54, 55]. Our plan for data safety monitoring will comprise investigators and study staff providing monthly presentations of study enrollment, protocol non-compliance, adverse events/harms {22}, and other activities such as protocol modifications to the University of Pittsburgh, Department of Emergency Medicine Departmental Clinical Research Meeting (DCRM). One faculty member and one IRB coordinator within the Department of Emergency Medicine of the University of Pittsburgh School of Medicine, who are unaffiliated with the study, will periodically audit trial conduct {23}.

### Criteria for discontinuing or modifying allocated interventions {11b}

Participation is voluntary; thus, participants may withdraw at any time for any reason, which may include self-reported inability to complete the study protocol as designed. Criteria for orderly discontinuing a participant’s involvement in the study include the following: (A) the participant voluntarily withdraws from the study, (B) the participant does not complete all procedures/visits per protocol, (C) the participant’s physician or one of the investigators feels that it is not in the best interest of the subject to be in the study (i.e., severe hypertension or hypotension), and (D) the participant’s BP exceeds a measurement value of 180/110 across multiple measures. In the event that a participant’s BP averages exceed the value of 180/110 while being measured in the presence of the study team, the study team will direct the participant to contact his/her healthcare provider to discuss the measures. There are no pre-defined criteria for modifying allocated interventions.

### Strategies to improve adherence to interventions {11c}

We will use the following strategies to improve adherence to study procedures: (A) address any and all concerns during the consenting procedure, (B) be flexible with study participants regarding scheduling (e.g., allow for protocol events during both weekdays and during the weekend), and (C) provide access to television, radio, and the Internet during all in-laboratory phases.

### Relevant concomitant care permitted or prohibited during the trial {11d}

During the study protocol, participants will be asked to not engage in scheduled or unscheduled shift work and avoid consumption of caffeine or alcohol and avoid nicotine. Two out of seven similar studies testing the impact of two different nap durations have allowed participants to consume one caffeinated beverage or smoke a cigarette at select points during the study protocol [56–66]. Caffeine is a stimulant that has a profound impact on subjective sleepiness and alertness [67] and therefore may impact the differences between study conditions. While previous research has assessed the interaction between caffeine and napping [68], that is not the focus of this protocol.

### Provisions for post-trial care {30}

All participants who may require medical attention will be provided medical attention at the University of Pittsburgh Medical Center, which is outlined in the consent form.

### Outcomes {12}

Our primary outcomes of interest include blunted BP dipping and reduced HRV as measured by the standard deviation of the inter-beat intervals of normal sinus beats (SDNN). Blunted BP dipping will be calculated using two measures. First, we will quantify sleep vs. wake BP dipping by identifying the participant’s sleep and wake times and stratifying the hourly ABPM measures into sleep vs. wake measures. The ratio of sleep-to-wake BP is calculated by taking the difference between the mean systolic and diastolic BP during the sleeping hours and the mean systolic and diastolic BP during waking hours. We define dipping status as follows: (mean wake hours BP − mean sleep BP divided by mean wake hours BP) × 100. Non-dipping status will be defined as sleep hours BP dip of less than 10%, assessed with systolic BP (SBP) and diastolic BP (DBP) [22, 69]. Next, we will stratify each 24-h period into nighttime vs. daytime periods and calculate nighttime BP dipping with the “wide fixed time method” [70, 71]. Daytime hours are defined as 0700 to 2259 h, and nighttime hours are defined as 2300 to 0659 h [70, 71]. The ratio of night-to-day BP is determined by taking the difference between the mean systolic and diastolic BP during the nighttime hours (2300 to 0659 h) and the mean systolic and diastolic BP during daylight hours (0700 to 2259 h). For this method, nighttime dipping status is determined as follows: ((mean daylight BP − mean nighttime BP) ÷ mean daylight hours BP) × 100. We will assess dipping vs. non-dipping status during sleep hours and during nighttime hours for all periods of observation and for all study conditions.

Our second outcome of interest is reduced HRV. As prescribed [72], we will examine HRV based on five time-based measures summarized over each 24-h period of observation. Measures include the following: (A) standard deviation of the inter-beat intervals of normal sinus beats (SDNN) summarized over 24 h; (B) standard deviation of the averaged normal sinus intervals for all 5-min segments (SDANN) summarized over 24 h; (C) the mean of the standard deviations of all normal sinus RR intervals for all 5-min segments (SDNN index) summarized over 24 h and stratified by wake, sleep, and work and non-work periods; (D) root-mean-square of the successive normal sinus RR interval difference (rMSSD) summarized over 24 h; and (E) the low-frequency (LF) band and high-frequency band (HF) ratio (LF/HF) summarized over 24 h and stratified by wake, sleep, and work and non-work periods. The LF/HF ratio is a frequency domain measure of HRV that depicts the balance between sympathetic and parasympathetic activity of the nervous system [41]. Low values reflect the dominance of the parasympathetic system whereas a high ratio is indicative of sympathetic dominance [41]. For medical risk stratification, the SDNN over a 24-h period is considered a gold standard measure [41]. Twenty-four-hour measures of SDNN < 50 ms are considered unhealthy [41, 73, 74].

We will administer multiple survey instruments at baseline and during the study period, including a standard demographics survey. At baseline, we will administer the Pittsburgh Sleep Quality Index (PSQI), which we will use to measure sleep quality [75]. The Epworth Sleepiness Scale (ESS) will be used to measure daytime sleepiness [76]. The Chalder Fatigue Questionnaire (CFQ) will measure mental and physical fatigue [77]. With permission from the developer, we will use the Occupational Fatigue and Recovery (OFER) Scale to assess acute fatigue, chronic fatigue, and inter-shift recovery [78]. The Copenhagen Burnout Inventory (CBI) will measure burnout [79]. Five items from the Schedule Attitudes Survey (SAS) will measure satisfaction with shift schedule [80].

Participants will wear a wrist-worn actigraph to objectively measure sleep/wake over the entire study period. We will also use data from these devices to estimate the body temperature minimum for each day of the study. Blood pressure and HRV vary by circadian phase, with a notable surge in morning BP after arousal [81]. As the sleep of shift workers in our study is dependent on the absence of emergency calls, sleep may be distributed across the day and night, and the magnitude of BP dip during sleep may depend on the participant’s circadian phase. When circadian rhythms are aligned with the sleep-wake cycle, the core body temperature minimum (CBT-min) usually occurs 2–3 h prior to the habitual wake time [82]. Data from actigraphy and photometry can be used to predict the timing of the CBT-min and determine circadian alignment/misalignment. The Circadian Performance Simulation Software (CPSS version 2.1, Brigham and Women’s Hospital, Boston, MA, USA) will be used to estimate the circadian phase for each sleep episode. This software was developed based on bio-mathematical models and has been utilized to characterize circadian rhythms of astronauts on the International Space Station [83–88]. Sleep vs. wake (binary) and light exposure data (binned in 1-h increments) collected from actigraphy and photometry will be used as inputs in the CPSS model. All available complete 24-h intervals of actigraphy and photometry data will be included in the analysis. The daily estimated endogenous circadian temperature minimum obtained from the CPSS program will be compared with the sleep episode time derived from actigraphy and photometry for that day. When the estimated endogenous circadian temperature minimum falls within a sleep episode, that sleep episode will be considered circadian “aligned.” When the estimated endogenous circadian temperature minimum falls outside the sleep episode, that day’s sleep episode will be considered to be circadian “misaligned.” The addition of circadian phase estimation will address a limitation that has been identified in previous research.

During the in-laboratory sleep periods (naps), we will use data from the Zmachine® Synergy (General Sleep Corporation, Cleveland, OH) to (A) measure stages of sleep and (B) explore the relationship between stages of sleep and changes in BP and HRV. We will also pose the question: Do EMS clinicians who obtain more time in deeper stages of sleep (i.e., stage 3 or 4) during intra-shift naps/sleep experience a deeper dip in BP (or a normal dip of 10–20%) than do clinicians who obtain more time in lighter stages of sleep (i.e., stages 1 or 2)?

### Participant timeline {13}

See Figs. 1 and 2 for the time schedule of enrollment, intervention, measurement assessment, and other relevant participant activities.

### Sample size {14}

Our goal enrollment is 35 total participants with an estimated 10 of the 35 enrolled not completing the study protocol or classified as lost to attrition. Our goal for enrollment is based on several factors. (A) Previous research suggests 10–33% [89, 90] of EMS workers will be willing to participate but excluded due to self-reporting a diagnosis of HTN and/or are currently taking medication for HTN. (B) Previous research suggests 10–15% attrition in prospective observational studies [19]. Attrition can impact inferential statistics if participants with complete follow-up data differ from participants who are lost to attrition. Our strategies to address attrition include the following: (A) provide participants with reasonable remuneration and (B) maintain regular communication with participants including, yet not limited to, regular email updates, telephone reminders, text message reminders, and other forms of frequent communication. We will compare available characteristics between those who cease participation and those who complete the study as designed.

### Recruitment {15}

We will recruit eligible participants from Western Pennsylvania in the USA. Western Pennsylvania is home to more than 300 EMS agencies and more than 9000 certified EMS clinicians [52]. All EMS agencies and EMS clinicians are licensed/certified by the state of Pennsylvania. Lists of EMS agencies and clinicians are maintained by regional offices and by health care systems that provide continuing education and other resources. We will use these lists to disseminate IRB-approved study flyers. In addition, we will circulate study-related flyers on social media and reach out to local EMS education programs to request study-related information be disseminated to trainees at all EMS levels of certification. If enrollment falls below two new participants per month, we will hold an online seminar/webinar and invite EMS clinicians in Western Pennsylvania to attend for purposes of learning more about the study and to ask questions.

### Sequence generation {16a}

An investigator not overseeing the in-lab sessions will construct the randomization sequence for each participant using the SAS statistical software procedure PROC PLAN (Cary, NC). The sequences will adhere to the Williams design [91], which ensures a uniform crossover design with a balance of order in the experiment as a whole.

### Concealment mechanism {16b}

The assignment for each participant visit will be transferred to sealed opaque envelopes to be opened at the beginning of each session.

### Implementation {16c}

Two previous studies with a similar study design reported concealing the objectives of the study and providing study participants with limited details of the intervention until moments before the intervention was administered [56, 59–61]. These studies show that full blinding of study participants and concealment of intervention details is difficult. Our protocol faces similar challenges. For purposes of our study, once the opaque envelope with randomization assignment is opened, investigators, study staff, and participants will be alerted to the assigned napping condition. Given this design, our study is open-label.

### Assignment of interventions: blinding

#### Who will be blinded {17a}

One member of the study team, the primary data analyst (statistician), will be blinded for the duration of the study. All other study investigators, staff, and participants will be blinded to the condition assignment immediately prior to each session. Once the envelope is opened, all who are on site at the time of randomization will be made aware of the assignment and unblinded.

#### Procedure for unblinding if needed {17b}

N/A. There are no procedures for unblinding once the opaque envelope has been opened.

### Data collection and management

#### Plans for assessment and collection of outcomes {18a}

The protocol for measurement assessment and collection of outcome measures includes the use of continuous monitoring devices, such as ABPM for serial BP measurement, and cardiac Holter monitoring for serial HRV measurement for the 72-h protocol repeated three times for each study participant. For ABPM, we will assess BP hourly with the Oscar2 device manufactured by SunTech Medical (Morrisville, NC, USA). For HRV, we will use the NASIFF CardioCard® 5-lead, 3-channel device (NASIFF Associates, Inc., Central Square, NY, USA). As described in the “Outcomes {12}” section, we will administer surveys at baseline and again at pre-specified time points in order to capture subjective ratings of sleep and fatigue. Our plan for assessment will also include monitoring of sleep depth during the conditions that involve intra-shift napping. We will use the Zmachine® Synergy for purposes of assessing depth of sleep during the in-laboratory phase only and only during the two napping conditions. Sleep versus wake will be assessed with a wrist-worn actigraphy device (wGT3X-BT by Actigraph Corp., Pensacolo, FL, USA) worn continuously during the three 72-h protocols.

Participants will complete standardized surveys at baseline, throughout the study protocol, and at the end of each 72-h period. At baseline, participants will be asked to complete a standard demographic questionnaire, the PSQI, ESS, CFQ, OFER, CBI, and SAS surveys. Study staff will complete seven standardized, paper-based data collection forms. These forms document the following: (A) the time when devices (i.e., wrist actigraphy, ABPM, Holter monitors) are applied and/or removed; (B) results of calibration of continuous monitoring devices (i.e., ABPM, Holter monitor, wrist actigraphy, and the Zmachine® Synergy). Calibration of the Oscar2 device will be similar to the process described in a separate publication [35]; (C) in-laboratory hourly, self-reported, single-item assessments of fatigue, sleepiness, irritability, stress, and related constructs analogous to the items used in previous research [92]; (D) in-laboratory, hourly measurements of psychomotor vigilance with the brief psychomotor vigilance test (PVT-B). The PVT-B is widely used to assess reaction time (vigilance and alertness), shown to be reliable, is sensitive to changes in sleep and sleep deprivation, resistant to practice effects, and considered a valid assessment of neurocognitive performance [93–96]; and (E) self-reported, single-item assessments of fatigue, sleepiness, irritability, stress, and related constructs [92] immediately upon waking from in-laboratory napping periods; again at 10 min, at 20 min, and at 30 min after waking from a nap. We will also document vigilance with the PVT-B immediately upon waking, at 10 min, 20 min, and 30 min. Study staff will transcribe the data documented on paper-based forms into a REDCap database immediately after a form is completed. A second study staff member will review the transcription for accuracy and completeness.

#### Plans to promote participant retention and complete follow-up {18b}

We will distribute a $400 gift card to each participant at the completion of each of the three study conditions. We chose $400 per study condition based on our prior success with recruitment and low attrition with the targeted population [19, 97, 98]. In total, each participant who completes the full study protocol will receive $1200 in remuneration. This amount is less than the total remuneration offered in previous studies with similar protocols and equivalent participant burden [56–58].

#### Data management {19}

All data will be downloaded from monitoring devices, or abstracted from survey instruments, and uploaded to a password-protected REDCap database maintained by the principal investigator’s institution [54, 55].

#### Confidentiality {27}

Confidentiality of study participation will be maintained by storing all paper-based data collection instruments and study-related forms in locked filing cabinets in the locked office of study investigators, located on the University of Pittsburgh campus. All data obtained from electronic devices will be assigned a code number, which will be used to then store participant data electronically in a password-protected REDCap database [54, 55].

#### Plans for collection, laboratory evaluation, and storage of biological specimens for genetic or molecular analysis in this trial/future use {33}

N/A this study protocol does not include the collection of biological specimens.

### Statistical methods

#### Statistical methods for primary and secondary outcomes {20a}

We will report the demographic characteristics of the study sample using descriptive statistics and stratify the final dataset into two categories for the main analysis. These categories will be labeled as “complete” and “incomplete data.” We will further describe the average systolic and diastolic BP, the incidence of blunted BP dipping, and the proportion of participants with blunted BP dipping. Blunted nocturnal BP dipping will be defined as previously described. Our second outcome of interest is HRV. As prescribed [72], we will examine HRV based on five time-based measures summarized over each 24-h period of observation.

To address the impact of napping on BP dipping, we will conduct paired *t* tests to determine if the mean amount of BP dipping differs across conditions. Generalized linear mixed models (GLMM) with participant-specific random effects will be utilized to compare the odds of blunted BP dipping across conditions. We will evaluate the distribution of residuals at each level of the models for linearity and heterogeneity. We will check for outliers and influential observations and sets of observations. The distribution of the data will be examined, and transformations will be performed as necessary for valid comparisons. We will use GLMM to estimate and compare the proportion of EMS workers with blunted BP dipping and control for the dependence between repeated measures within each participant with a random subject effect.

To address the impact of napping on select measures of HRV, we will use GLMM with appropriate distribution and link function to estimate and compare HRV measures between EMS workers across the three conditions. We will again account for the within-participant correlation of repeated measurements and any participant-dependent covariates that vary in the study sample. The least square means and corresponding 95% confidence intervals will be calculated from the models for each group and the difference between groups.

We also plan to compare the slope of HRV and BP amplitude following the simulated shift work across the three conditions. The hypothesis testing will again follow the GLMM framework. Time-varying slopes will be calculated for each participant for the recovery period. These slopes will characterize the change in the outcome measures (HRV and BP amplitude) following shift work. We will account for the within-participant correlation of repeated measurements and any participant-dependent and time-dependent covariates. We will account for actual sleep during the periods of nap opportunity by running multiple statistical models with one set of models standardized for the nap opportunity (i.e., 30 min vs. 120 min) and another set of models that account for the depth of sleep (stage) and the amount of time within each stage of sleep as measured by the Zmachine® Synergy.

Enrollment of 25 participants is projected to provide 80% power to detect a difference in the mean BP dip of 5 mmHg between conditions, assuming two-sided tests, alpha set at 0.05, and a within-participant standard deviation of 6 mmHg (similar to our previous work). Power was calculated using G*Power V3.1.9.2, using two-sided *z* tests in a Poisson regression with alpha set at 0.05.

#### Interim analyses {21b}

N/A. This study does not include an interim analysis.

#### Methods for additional analyses (e.g., subgroup analyses) {20b}

N/A. This study does not include a pre-defined list of participant demographic characteristics for purposes of subgroup analysis.

#### Methods in analysis to handle protocol non-adherence and any statistical methods to handle missing data {20c}

Participants with < 70% of scheduled ABPM measures or incomplete data capture (< 70%) with other measures of interest will be classified as “incomplete.” These data will be analyzed separately from participants with complete data.

#### Plans to give access to the full protocol, participant-level data, and statistical code {31c}

Study-related materials and study-related data would be made available upon request and with permission and approval from the University of Pittsburgh. All requests must be reasonable.

### Oversight and monitoring

#### Composition of the coordinating center and trial steering committee {5d}

This study does not include a coordinating center. The study principal investigator and two co-investigators will be responsible for monitoring and managing data quality, assess completeness and accuracy of data collection, implementation and adherence to the study protocol, and measurement of outcomes.

#### Composition of the data monitoring committee, its role, and reporting structure {21a}

Study-related progress and data (e.g., enrolment, withdrawals) will be reported monthly to the University of Pittsburgh, Department of Emergency Medicine Departmental Clinical Research Meeting (DCRM). The DCRM comprised the department’s vice chair of research, senior faculty, study coordinators, and department liaisons with the university’s institutional review board. The members receive monthly updates, provide feedback to investigators, and advise on study-related challenges and events that may have been or need to be reported to the university’s institutional review board and/or funding organization.

#### Frequency and plans for auditing trial conduct {23}

All data maintained electronically in the REDCap database will be examined for accuracy (quality) and reported during the monthly DCRM meetings.

#### Plans for communicating important protocol amendments to relevant parties (e.g., trial participants, ethical committees) {25}

All protocol modifications or amendments will be reported (submitted) to the University of Pittsburgh Institutional Review Board. Participants will be notified of the amendments or modifications that impact participation, confidentiality, or safety.

#### Dissemination plans {31a}

Our plan for dissemination includes peer-reviewed manuscripts and presentations at professional meetings.

## Discussion

Paramedics and EMTs are essential front-line public safety workers who face excess risk of workplace injury, mental stress, fatigue, poor sleep quality, and poor health and well-being. The workforce is limited and poorly compensated, which is evidenced by a large proportion working multiple jobs or excessive hours of overtime. There is great uncertainty regarding the optimal duration of an intra-shift nap, how best to implement a napping program, and how napping may impact performance and worker health. Our study will address worker health outcomes, specifically two indicators of cardiovascular health, and determine if shorter or longer duration naps during night shifts have a clinically meaningful impact. Our study will also assess recovery of these indicators immediately post-night shift, which will provide additional information for decision-makers and individual EMS workers.

The experimental protocol outlined in this paper is novel for the following reasons: (A) it builds on recent evidence-based guidelines that promote intra-shift napping for fatigue mitigation, (B) the study will involve EMS clinicians as study subjects and therefore provide much needed direct evidence as opposed to indirect evidence involving “non-EMS” volunteers, and (C) our outcomes of interest include key indicators of cardiovascular disease. With few having assessed these outcomes in this population [99], under these conditions, the results of this study will provide new insights for researchers and additional evidence to guide decision-making.

One key component of our study is the comparison of the shorter (30 min) nap to the longer (2 h) nap. There are relatively few studies comparing shorter and longer duration naps [57, 62], and even less information about nap duration germane to EMS work [50]. Many in public safety would likely favor shorter duration naps given the potential for sleep inertia and how it can negatively impact performance immediately upon waking [8, 51]. The favorability of longer-duration naps is unknown. However, data from our recent observational study of 56 EMS night shift workers shows that longer duration naps (e.g., > 60 min) offer greater benefits to cardiovascular health than shorter naps in the form of a healthy dip in BP [35]. The study protocol outlined in this paper will help clarify the differences and similarities of different nap durations for EMS workers. It is plausible that we discover shorter duration naps are nearly as beneficial as longer duration naps for key indicators of cardiovascular health. It is also equally plausible that we discover longer duration naps are needed in order to observe benefit in the form of a healthy dip in BP. Regardless of our findings, the results will be informative to local EMS employers and decision-makers responsible for fatigue mitigation and workplace wellness.

## Trial status

Protocol version 1 as of February 17, 2021. Recruitment is scheduled to commence in March of 2021. The final participants are expected to complete the study at the end of the calendar year 2022.

## Supplementary Information

**Additional file 1:.** SPIRIT 2013 Checklist: Recommended items to address in a clinical trial protocol and related documents.

**Additional file 2:.** Consent to act as a subject in a research study.

## Abbreviations

ABPM: Ambulatory blood pressure monitoring
BP: Blood pressure
CBT: Core body temperature
CFQ: Chalder Fatigue Questionnaire
CPSS: Circadian Performance Simulation Software
CVD: Cardiovascular disease
DBP: Diastolic blood pressure
DCRM: Departmental Clinical Research Meeting
EMS: Emergency medical services
EMT: Emergency medical technician
ESS: Epworth Sleepiness Scale
GLMM: Generalized linear mixed models
HF: High-frequency band
HRV: Heart rate variability
HTN: Hypertension
IRB: Institutional Review Board
LF: Low-frequency band
MA: Massachusetts
NC: North Carolina
OFER: Occupational Fatigue and Recovery Scale
OH: Ohio
PSQI: Pittsburgh Sleep Quality Index
PVT: Psychomotor Vigilance Test
REDCap: Research Electronic Data Capture
rMSSD: Root-mean-square of the successive normal sinus RR interval difference
SAS: Schedule Attitudes Survey
SBP: Systolic blood pressure
SDNN: Standard deviation of the inter-beat intervals of normal sinus beats
SDANN: Standard deviation of the averaged normal sinus intervals for all 5-min segments
USA: United States of America
