# Supplementary material for: Napping on the night shift and its impact on blood pressure and heart rate variability among emergency medical services workers: study protocol for a randomized crossover trial
Source: Trials. 2021 Mar 16;22:212. doi: 10.1186/s13063-021-05161-4 (PMC7962082; doi:10.1186/s13063-021-05161-4)
Supplement: Supplementary file 1 — Additional file 1:. SPIRIT 2013 Checklist: Recommended items to address in a clinical trial protocol and related documents. [file 13063_2021_5161_MOESM1_ESM.doc]

**Additional file 1: SPIRIT 2013 Checklist: Recommended items to address in a clinical trial protocol and related documents**

| Section/  item | Item No | Description | Location in Paper |
| --- | --- | --- | --- |
| **Administrative information** | | |  |
| Title | 1 | Descriptive title identifying the study design, population, interventions, and, if applicable, trial acronym | Page 1  Title Page and reported within the Administrative Information table appearing on Page 2 of the document beneath the abstract. |
| Trial registration | 2a | Trial identifier and registry name. If not yet registered, name of intended registry | Referenced in the abstract and in the Trial Design section {8}. Page 9 of main document. |
|  | 2b | All items from the World Health Organization Trial Registration Data Set | All items are reported in the clinicaltrials.gov registration, Appendix A, and within the main document. |
| Protocol version | 3 | Date and version identifier | Referenced in the Trial Status section {8} of the main document. Page 9. |
| Funding | 4 | Sources and types of financial, material, and other support | Title page, page 1, and in the Declarations section on page 30 of main document. |
| Roles and responsibilities | 5a | Names, affiliations, and roles of protocol contributors | Title page, page 1, and on page 31-32 of main document. |
|  | 5b | Name and contact information for the trial sponsor | Title page, page 1, and on page 32 of main document. |
|  | 5c | Role of study sponsor and funders, if any, in study design; collection, management, analysis, and interpretation of data; writing of the report; and the decision to submit the report for publication, including whether they will have ultimate authority over any of these activities | Title page, page 1, and on page 30 of the main document. |
|  | 5d | Composition, roles, and responsibilities of the coordinating centre, steering committee, endpoint adjudication committee, data management team, and other individuals or groups overseeing the trial, if applicable (see Item 21a for data monitoring committee) | No coordinating center. Other components of 5d are reported in {5d} and {21a} of main paper on pages 26-27. |
| Introduction |  |  |  |
| Background and rationale | 6a | Description of research question and justification for undertaking the trial, including summary of relevant studies (published and unpublished) examining benefits and harms for each intervention | Background section of paper; pages 5-7 of main document. |
|  | 6b | Explanation for choice of comparators | Pages 5-8 of main document and pages 11-12 of main document. |
| Objectives | 7 | Specific objectives or hypotheses | Page 8 of main document. |
| Trial design | 8 | Description of trial design including type of trial (eg, parallel group, crossover, factorial, single group), allocation ratio, and framework (eg, superiority, equivalence, noninferiority, exploratory) | Pages 8-9 of main document. |
| Methods: Participants, interventions, and outcomes | | |  |
| Study setting | 9 | Description of study settings (eg, community clinic, academic hospital) and list of countries where data will be collected. Reference to where list of study sites can be obtained | Pages 9-10 of main document. |
| Eligibility criteria | 10 | Inclusion and exclusion criteria for participants. If applicable, eligibility criteria for study centres and individuals who will perform the interventions (eg, surgeons, psychotherapists) | Pages 10-11 of main document. |
| Interventions | 11a | Interventions for each group with sufficient detail to allow replication, including how and when they will be administered | Pages 12-15 of main document. |
|  | 11b | Criteria for discontinuing or modifying allocated interventions for a given trial participant (eg, drug dose change in response to harms, participant request, or improving/worsening disease) | Page 15 of main document. |
|  | 11c | Strategies to improve adherence to intervention protocols, and any procedures for monitoring adherence (eg, drug tablet return, laboratory tests) | Page 16 of main document. |
|  | 11d | Relevant concomitant care and interventions that are permitted or prohibited during the trial | Page 16 of main document. |
| Outcomes | 12 | Primary, secondary, and other outcomes, including the specific measurement variable (eg, systolic blood pressure), analysis metric (eg, change from baseline, final value, time to event), method of aggregation (eg, median, proportion), and time point for each outcome. Explanation of the clinical relevance of chosen efficacy and harm outcomes is strongly recommended | Pages 16-19 of main document. |
| Participant timeline | 13 | Time schedule of enrolment, interventions (including any run-ins and washouts), assessments, and visits for participants. A schematic diagram is highly recommended (see Figure) | Section {13} on page 19 of main document. See figures 1 and 2. |
| Sample size | 14 | Estimated number of participants needed to achieve study objectives and how it was determined, including clinical and statistical assumptions supporting any sample size calculations | Section {14} starting on pages 19-20 of main document. |
| Recruitment | 15 | Strategies for achieving adequate participant enrollment to reach target sample size | Offering remuneration for participation (see paragraph # 17 of the Methods section. Offering paid transportation at different phases of the protocol – as described paragraph #7 of the Methods section. |
| **Methods: Assignment of interventions (for controlled trials)** | | |  |
| Allocation: |  |  |  |
| Sequence generation | 16a | Method of generating the allocation sequence (eg, computer-generated random numbers), and list of any factors for stratification. To reduce predictability of a random sequence, details of any planned restriction (eg, blocking) should be provided in a separate document that is unavailable to those who enroll participants or assign interventions | Page 21 of main document. |
| Allocation concealment mechanism | 16b | Mechanism of implementing the allocation sequence (eg, central telephone; sequentially numbered, opaque, sealed envelopes), describing any steps to conceal the sequence until interventions are assigned | Page 21 of main document. |
| Implementation | 16c | Who will generate the allocation sequence, who will enroll participants, and who will assign participants to interventions | Page 21 of main document in section {16c}. |
| Blinding (masking) | 17a | Who will be blinded after assignment to interventions (eg, trial participants, care providers, outcome assessors, data analysts), and how | Section {17a} on page 21 of main document. |
|  | 17b | If blinded, circumstances under which unblinding is permissible, and procedure for revealing a participant’s allocated intervention during the trial | Section {17b} on page 22 of main document. |
| **Methods: Data collection, management, and analysis** | | |  |
| Data collection methods | 18a | Plans for assessment and collection of outcome, baseline, and other trial data, including any related processes to promote data quality (eg, duplicate measurements, training of assessors) and a description of study instruments (eg, questionnaires, laboratory tests) along with their reliability and validity, if known. Reference to where data collection forms can be found, if not in the protocol | Pages 22-23 of main document. |
|  | 18b | Plans to promote participant retention and complete follow-up, including list of any outcome data to be collected for participants who discontinue or deviate from intervention protocols | Page 23 of main document in section {18b}. |
| Data management | 19 | Plans for data entry, coding, security, and storage, including any related processes to promote data quality (eg, double data entry; range checks for data values). Reference to where details of data management procedures can be found, if not in the protocol | Section {19} on pages 23-24 of main document. |
| Statistical methods | 20a | Statistical methods for analysing primary and secondary outcomes. Reference to where other details of the statistical analysis plan can be found, if not in the protocol | Section {20a} on pages 24-25 of main document. |
|  | 20b | Methods for any additional analyses (eg, subgroup and adjusted analyses) | Section {20b} on page 26 of main document. |
|  | 20c | Definition of analysis population relating to protocol non-adherence (eg, as randomised analysis), and any statistical methods to handle missing data (eg, multiple imputation) | Section {20c} on page 26 of main document. |
| **Methods: Monitoring** | | |  |
| Data monitoring | 21a | Composition of data monitoring committee (DMC); summary of its role and reporting structure; statement of whether it is independent from the sponsor and competing interests; and reference to where further details about its charter can be found, if not in the protocol. Alternatively, an explanation of why a DMC is not needed | Section {21a} on page 27 of main document. |
|  | 21b | Description of any interim analyses and stopping guidelines, including who will have access to these interim results and make the final decision to terminate the trial | Section {21b} on page 26 of main document. |
| Harms | 22 | Plans for collecting, assessing, reporting, and managing solicited and spontaneously reported adverse events and other unintended effects of trial interventions or trial conduct | See page 15 of main document. |
| Auditing | 23 | Frequency and procedures for auditing trial conduct, if any, and whether the process will be independent from investigators and the sponsor | See page 15 of main document. |
| Ethics and dissemination | | |  |
| Research ethics approval | 24 | Plans for seeking research ethics committee/institutional review board (REC/IRB) approval | Page 9 of main document. |
| Protocol amendments | 25 | Plans for communicating important protocol modifications (eg, changes to eligibility criteria, outcomes, analyses) to relevant parties (eg, investigators, REC/IRBs, trial participants, trial registries, journals, regulators) | Section {25} on page 27 of main document. |
| Consent or assent | 26a | Who will obtain informed consent or assent from potential trial participants or authorised surrogates, and how (see Item 32) | Section {26a} on page 11 of main document. |
|  | 26b | Additional consent provisions for collection and use of participant data and biological specimens in ancillary studies, if applicable | Section {26b} on page 11 of main document. |
| Confidentiality | 27 | How personal information about potential and enrolled participants will be collected, shared, and maintained in order to protect confidentiality before, during, and after the trial | Section {27} on page 24 of main document. |
| Declaration of interests | 28 | Financial and other competing interests for principal investigators for the overall trial and each study site | Section {28} on page 31-32 of main document. |
| Access to data | 29 | Statement of who will have access to the final trial dataset, and disclosure of contractual agreements that limit such access for investigators | Section {29} on page 31 of main document. |
| Ancillary and post-trial care | 30 | Provisions, if any, for ancillary and post-trial care, and for compensation to those who suffer harm from trial participation | Section {30} on page 16 of main document. All participants who may require medical attention will be provided medical attention at the University of Pittsburgh Medical Center. This is outlined in the consent form. |
| Dissemination policy | 31a | Plans for investigators and sponsor to communicate trial results to participants, healthcare professionals, the public, and other relevant groups (eg, via publication, reporting in results databases, or other data sharing arrangements), including any publication restrictions | Section {31a} on page 27 of main document. |
|  | 31b | Authorship eligibility guidelines and any intended use of professional writers | Section {31b} on page 30 of main document. |
|  | 31c | Plans, if any, for granting public access to the full protocol, participant-level dataset, and statistical code | Section {31c} and {29} on page 31 of main document. |
| Appendices |  |  |  |
| Informed consent materials | 32 | Model consent form and other related documentation given to participants and authorised surrogates | See Appendix B |
| Biological specimens | 33 | Plans for collection, laboratory evaluation, and storage of biological specimens for genetic or molecular analysis in the current trial and for future use in ancillary studies, if applicable | Section {33} on page 24 of main document. |

*It is strongly recommended that this checklist be read in conjunction with the SPIRIT 2013 Explanation & Elaboration for important clarification on the items. Amendments to the protocol should be tracked and dated. The SPIRIT checklist is copyrighted by the SPIRIT Group under the Creative Commons “[Attribution-NonCommercial-NoDerivs 3.0 Unported](http://www.creativecommons.org/licenses/by-nc-nd/3.0/)” license.
