# Supplementary material for: Napping on the night shift and its impact on blood pressure and heart rate variability among emergency medical services workers: study protocol for a randomized crossover trial
Source: Trials. 2021 Mar 16;22:212. doi: 10.1186/s13063-021-05161-4 (PMC7962082; doi:10.1186/s13063-021-05161-4)
Supplement: Supplementary file 2 — Additional file 2:. Consent to act as a subject in a research study. [file 13063_2021_5161_MOESM2_ESM.pdf]

University of Pittsburgh

Department of Emergency Medicine
3600 Forbes Avenue
Iroquois Bldg., Suite 400A
Pittsburgh, PA 15261

CONSENT TO ACT AS A SUBJECT IN A RESEARCH STUDY

TITLE: What is the impact of napping during night shifts on indicators of cardiovascular health among EMS clinicians?

PRINCIPAL INVESTIGATOR	Co-Investigator (primary contact)
Leonard Weiss, MD<br>University of Pittsburgh<br>School of Medicine<br>Department of Emergency Medicine<br>weissls2@upmc.edu	Daniel Patterson, PhD, NRP<br>University of Pittsburgh<br>School of Medicine<br>Department of Emergency Medicine<br>pdp3@pitt.edu<br>(412)-843-3830

KEY INFORMATION:

- You are being asked to take part in a research study. Research Studies include only people who choose to take part. The study team members will explain the study to you and will answer any questions you might have. You should take your time to make your decision.
- The goal of this study is to understand how napping during night shift can potentially affect indicators of cardiovascular health among EMS workers.
- You will be asked to participate in three 72-hour arms, the first 24 hours occurring at home, then 24 hours in the lab, and finally the last 24 hours at home again.
- You will wear monitors that measure various physiologic activity and will be asked to log your sleep, perform psychomotor evaluations, as well as a simulated EMS night shift.
- You will be eligible for $400 remuneration for each 72-hour study arm you complete.
- There will be no painful or invasive procedures involved in this study. You may experience discomfort associated with sleep deprivation or repeated activity completion. Rarely, there is a risk that your personal information would be exposed if there was a data breach, although many factors are in place to prevent this from happening.

SOURCE OF SUPPORT: This study is supported by a grant from the ZOLL Foundation.

Why is this research being done?

This study will help us to determine the impact of napping during night shifts on key indicators of cardiovascular health among EMS clinician shift workers. The key indicators of interest include blood pressure (BP) and heart rate variability (HRV). Abnormal patterns of BP and HRV are widely accepted indicators of poor cardiovascular health. Previous research shows that many

who are exposed to shift work conditions exhibit abnormal patterns of BP and HRV. Several observations have led us to propose this study and to focus on napping during night shifts.

First, previous research shows that 2-3 days of rest post shift work may be necessary before BP and HRV normalize.

Second, our recent research shows that napping during night shifts may be helpful with respect to BP.

Our ultimate goal is to determine if napping during night shift has an impact on BP and HRV among Emergency Medical Services (EMS) shift workers. These data will inform or guide the development of interventions for shift workers that may help mitigate these abnormalities and the downstream impact on cardiovascular health.

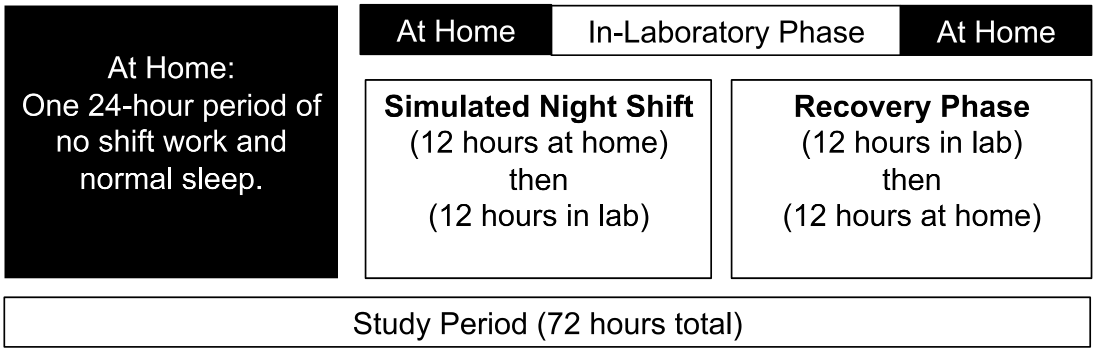

Who is being asked to take part in this research study?
We are asking for the voluntary participation of certified EMS clinicians (e.g., EMTs, paramedics, flight nurses/paramedics), and other EMS clinicians that work as EMS clinicians in Western Pennsylvania over the age of 18. All who voluntarily participate should be without significant health issues and without health concerns that may affect their blood pressure or heart rate variability. We hope to enroll about 35 participants.

What procedures will be performed for research purposes?
If you decide to take part in this research study, you will be asked to complete three study arms that last approximately 72 continuous hours each. See the figure here below for a graphical illustration. We will now explain all procedures.

We will ask you complete three study arms.

Each study arm will involve the following procedures:

1] You will come into the Applied Physiology Lab (APL) located at the University of Pittsburgh Deparmtent of Emergency Medicine's research offices in the Iroquois Building Suite 400A and arrive between 0500 and 0600 hours. You will be screened to determine if you have any signs or symptoms of COVID19 prior to entering the laboratory. We will ask you questions about recent fever or chills, cough, shortness of breath, body fatigue, muscle or body aches, headache, new loss of taste or smell, sore throat, congestion or runny nose, nausea, vomiting, or diarrhea. We will also take your temperature. If you answer yes to any of these screening question and/or if your temperature is 100.3 degrees Fahrenheit or 37.9 degrees Celsius, we will consult an emergency physician who is involved with the study protocol to determine if you can continue or should reschedule.

2] You will asked to complete multiple paper-based surveys throughout the study.

3] You will be asked to wear an ambulatory blood pressure (ABPM) monitoring device on your left upper arm. This ABPM device will measure your BP every hour.

4] You will be asked to wear a Holter monitor that measures your heart rate and heart rate variability (HRV).

5] You will be asked to wear a wrist-worn Actigraph device that measures your sleep and wake activity.

6] You will be asked to complete a paper-based sleep diary.

7] During this at-home phase of the study, we will ask that you do not work at all (no shift work) and comply with a strict bedtime of (2200 hours) and wake time of (0800 hours). While you are at-home, we will collect data with various monitoring devices and communicate with you via mobile phone text message to alert you with reminders.

8] After the first 24-hours of starting each study arm, we will ask that you return to the APL lab around 1700 or 1800 hours so you can start a planned 12-hour simulated night shift. Once you arrive in the lab, we will randomize you to one of three study arms ([1] the no nap study arm, [2] the brief 30 minute nap opportunity study arm; or the [3] 2-hour nap opportunity study arm). During the simulated night shift, you will be asked to complete a variety of EMS-related tasks such as simulated dispatches and patient care simulations. Our study team will lead the simulations.

9] While you are in the lab, we will ask you to complete a psychomotor vigilance test (PVT) every hour with our mobile iPad device. All PVTs involve completing a 3-minute reaction time assessment. The PVT assessment is commonly used in sleep related studies.

10] If at the start of a study are you are randomized to one of the two napping study arms, we will have you wear a portable sleep stage monitoring device (The Zmachine® Synergy) for the napping opportunity period only. This device will help us determine what stage of sleep you achieve during the sleep opportunity.

11] After your 12-hour simulated night shift is over (around 0700), you will then enter the recovery phase and remain in the APL laboratory until 1900 hours. You will remain awake except for a nap allowed between 13:00-15:00. You will continue to wear the portable sleep stage monitoring device, and we will continue to monitor your BP, heart rate, sleep/wake status, and ask you to complete PVT assessments every hour, and answer various paper-based surveys.

12] At approximately 1900 hours of the in-lab recovery phase, we will end the in-lab phase and escort you home with a taxi or rideshare service. You will be sent home with the blood pressure, heart rate, wrist actigraphy, and a paper-based sleep diary. You will be asked to avoid any work-related activity (e.g., shift work) and focus on recovery sleep for the next 12 hours. We will ask that you comply with a strict bedtime of (2200 hours) and wake time of (0800 hours).

13] On the following day, we ask that you return to the APL lab to complete the study arm protocol. We ask that you arrive between 0800 and 0900 hours. We will then collect the study devices and documents. At this time we will provide you with $400 remuneration in the form of a University of Pittsburgh approved gift card. If you require assistance returning home, we will then escort you back home with a taxi or rideshare service. We will also schedule the next time you will be involved in the study so you may complete the next two study arms.

14] During all phases of the study (each 72-hour arm), caffeine use will be prohibited. Fatigue or other affects associated withdrawal may be experienced if you are a heavy user. During the in-lab phase of the study, your caloric intake will consist of scheduled meals and snacks based off reasonable caloric intake levels. Meal and snack offerings will be provided by the study team.

15] In response to COVID-19, we will ask that all study participants wear a face mask while in the laboratory (inside the University building) and while participating in the study during the laboratory phase. You do not have to wear a facemask while completing the at-home components of this protocol. Participants will be allowed to take off the facemask when performing the in-laboratory napping (sleeping) component of the protocol.

Feedback
Following completion of all 3-study arms, we will provide you with a 1-page summary of your blood pressure measures over the study period.

What are the possible risks, side effects and discomforts of this research study?

The risk of completing the surveys/questionnaires, and diaries are minimal but the nature of some of the questions is personal (for example, about your health status and well-being) and answering them might be stressful, uncomfortable, frustrating, or embarrassing. You are not obligated to answer any questions that make you uncomfortable.

The risk of participating in simulated dispatched EMS cases may entail being suddenly stimulated from a resting or sleeping state, which is likely temporarily uncomfortable. Also minimal physical activity such as bending, positioning, using equipment that would represent

typical medical care activities on an EMS call. Fatigue associated with working a night shift may be experienced. We will not employ any excessive lifting or strenuous activity.

The ambulatory blood pressure monitoring device (ABPM) is worn on your upper left arm and will take a blood pressure once every hour during each observation period. These devices are commonly used in healthcare settings. The repeated arm cuff inflations can cause some minor discomfort and can cause some temporary bruising. You may experience similar discomfort with the wrist-worn blood pressure monitoring device, and the device may cause you to wake from sleep. You may remove it when showering or doing other personal activities, however, we request that you wear it continuously and limit the amount of time you do not wear it.

The wristwatch actigraph monitors are worn on the wrist. The armband and wristband secure the device to you. These devices are often tolerated well by most participants; however, you may experience some skin irritation or discomfort. You may also remove the device when showering or performing other personal activities, however, we request that you wear it continuously and limit the amount of time you do not wear it.

The heart rate variability monitor is a 5-electrode monitor that will be worn on your chest. The electrodes are attached to the participant via 5 electrode stickers, which may cause minor discomfort and/or skin irritation. You may also remove the device/electrodes when showering or performing other personal activities, however, we request that you wear it continuously and limit the amount of time you do not wear it.

PLEASE know that if you take a shower or bath, you will need to remove the devices listed above. Please do not wear these devices while exposed to water.

Please also avoid exercising during the 72 hours of continuous observation.

When you are in the lab and are scheduled to wear the the Zmachine® Synergy sleep monitoring device, you may experience discomfort. The device requires you wear a finger-positioned pulse oximetry monitor, a nasal cannula, a chest strap to hold the monitoring device, and three electrode stickers on the back of your neck/head. Wearing the device may case irritation or discomfort. The electrode stickers may cause minor discomfort and/or skin irritation.

The iPad psychomotor vigilance test (PVT) will be administered every hour during the in-laboratory phase. Use of the iPad and PVT tool will require that you tap the screen of the tablet computer repeatedly over a period of three minutes. This tapping motion may cause some mild irritation to the end of your finger; however, the risk is small and most do not report any problems. This process may be bothersome or irritating to some people. Tapping on the screen of the device can cause some minor discomfort.

You will be asked to complete a paper-based sleep diary during the at-home phases of the study protocol. Completing the paper-based surveys for this project will require use of a pencil or pen and several minutes to complete. Some may become dissatisfied or frustrated with the time required to complete the documents.

In terms of risk related to data, only research associates are involved in this study protocol will have access to your data collected from the various devices and instruments. No research findings will be provided to your family members, insurance companies, employers, or any third party without your authorization.

An accidental or inadvertent breach of confidentiality is a potential risk of participation. Most study materials are identified with an ID number only. We will collect personal identifiable information, such as your name, telephone number on several study forms. These materials will be kept securely locked in a filing cabinet located in the principal investigator's locked office on the University of Pittsburgh campus. All electronic materials will be kept on password protected computer servers accessed only by the study team. The PVT assessment data are coded and cannot be linked to other study information by anyone other than the study team. All devices used to collect your PVT data are password protected and the data are not kept on the device. The data captured on the iPad device will be stored on a secure database accessed only by the study team. The dataset for analysis purposes will be kept on protected computers located on the University of Pittsburgh campus, and maintained on the University of Pittsburgh and University of Pittsburgh Medical Center's computer security systems.

All data will be de-identified upon completion of the study and kept indefinitely for research purposes. We will attempt to publish the study findings in aggregate in peer-reviewed medical journals. We will do everything possible to protect your privacy, but there is always a risk that confidentiality could be breached. Individuals not affiliated with this study could learn information about you if confidentiality is breached. This breach could impact your employment or health insurance, or could lead to discrimination or family relationship problems, or other. Safeguards to protect your confidentiality are discussed below under the heading "Who will know about my participation in this research study?"

What are the possible benefits of taking part in this research study?

You may receive no direct benefit from taking part in this research study. However, you will learn about your blood pressure and heart rate variability in relation to simulated night shifts and recovery from night shifts, which may affect your risk for chronic disease. You will be notified of any ambulatory blood pressure results or abnormal heart rate variability results that require immediate attention (for example, high blood pressure) and referred for appropriate treatment.

Will I, or my insurance provider be charged for the costs of any procedures performed as part of this research study?

There are no costs to you or your insurance carrier for participating in this study.

Will I be paid if I take part in this research study?

Each participant will receive $400 in remuneration at the end of each 72-hour study arm. Participants who complete all 3-study arms will receive $1,200 in total remuneration.

Participants must complete a 72-hour study arm in order to receive the $400 remuneration. Early withdrawal from the research study (failure to complete all study arms) will be associated with you receiving less that the total remuneration. We also provide participants with a challenge coin, coffee mug, and EMS patches with University of Pittsburgh, Department of Emergency Medicine and EMS specific logos.

We will provide you with a parking voucher when you park onsite. When necessary, we will escort you home and/or to the facility with a taxi or rideshare program. If you leave your vehicle on site and need to be reunited with your vehicle, we will arrange transportation with a taxi or rideshare program.

Since you are being compensated for your participation in this study, your name, address, and social security number will be released to the Accounting Office. If the total reimbursement for your participation in research is greater than $600 in a year, this will be reported to the Internal Revenue Service (IRS) as income.

Who will pay if I am injured as a result of taking part in this research study?

University of Pittsburgh researchers and their associates who provide services at University of Pittsburgh Medical Center (UPMC) recognize the importance of your voluntary participation in their research studies. These individuals and their staffs will make reasonable efforts to minimize, control, and treat any injuries that may arise as a result of this research. *If you believe you have been injured as a result of the procedures that are performed for research purposes, immediately contact the Principal Investigator or one of the researchers listed on the first page. Emergency medical treatment for injuries solely and directly related to your participation in this research study will be provided by UPMC hospitals. If you do not have access to a UPMC facility, you should seek emergency care from your local hospital and call the University of Pittsburgh Human Subject Protection Advocate (1-866-212-2668). It is possible that UPMC or our local hospital may bill your insurance provider for the costs of this emergency treatment, but none of these costs will be charged directly to you. If your research-related injury requires medical care beyond this emergency treatment, you will be responsible for the costs. At this time there is no plan for additional financial compensation. You do not, however, waive your legal rights by signing this form.*

Who will know about my participation in this research study?

We will not report individual results to anyone not directly involved in this study. This study is being conducted at the University of Pittsburgh Department of Emergency Medicine where all staff are accustomed to dealing with personally sensitive information. Staff are trained regarding the critical nature of participants' privacy, and about the procedures for respecting their privacy and maintaining confidentiality. Data will be stored in locked areas immediately after collection and staff are instructed never to discuss participants' behavior outside the research study, and never to mention names of participants except to our research staff on the project. Your name will appear on the screening form along with your cellular telephone number and email. Other documents will use a study ID number. This same ID# will be used for storing data on computer; this information will generally consist of numbers with no meaning to the casual observer. You will not be identified by name in any publication of the research results unless you sign a separate form giving your permission (release).

What uses of my identifiable medical record information will this research study involve?
This research study will not involve the use or disclosure of your identifiable medical information.

Who will have access to identifiable information related to my participation in this research study?
In addition to the investigators listed on the first page of this authorization (consent) form and their research staff, the following individuals will or may have access to identifiable information related to your participation in this research study:

Authorized representatives of the University of Pittsburgh Office of Research Protections may review your identifiable research information for the purpose of monitoring the appropriate conduct of this research study.

We may share de-identified data with investigators conducting similar research or for educational purposes.

In unusual cases, the investigators may be required to release identifiable information related to your participation in this research study in response to an order from a court of law. If the investigators learn that you or someone with whom you are involved is in serious danger or potential harm, they will need to inform, as required by Pennsylvania law, the appropriate agencies.

A description of this clinical trial will be available on http://www.clinicaltrials.gov, as required by US Law. This website will not include information that can identify you. At most, the website will include a summary of the results. You can search this website at any time.

For how long will the investigators be permitted to use and disclose identifiable information related to my participation in this research study?

The investigators may continue to use and disclose, for the purposes described above, identifiable information related to your participation in this research study for a period of at least seven years after the end of the study and analysis of study data.

The research information that we collect from you will be labeled with an assigned code number and nothing that personally identifies you will be associated with this research information. This de-identified coded research information may be shared with other researchers for an indefinite period of time.

Is my participation in this research study voluntary?

Your participation in this research study, to include the use and disclosure of your identifiable information for the purposes described above, is completely voluntary. (Note, however, that if you do not provide consent for the use and disclosure of your identifiable information for the purposes described above, you will not be allowed to participate in the research study.) Whether or not you provide your consent for participation in this research study will have no effect on your current or future relationship with the University of Pittsburgh. Whether or not you provide your consent for participation in this research study will have no effect on your current or future medical care at a UPMC hospital or affiliated health care provider or your current or future relationship with a health care insurance provider.

May I withdraw, at a future date, my consent for participation in this research study?

You may withdraw, at any time, your consent for participation in this research study, to include the use and disclosure of your identifiable information for the purposes described above. (Note, however, that if you withdraw your consent for the use and disclosure of your identifiable information for the purposes described above, you will also be withdrawn, in general, from further participation in this research study). Any identifiable research recorded for, or resulting from, your participation in this research study prior to the date that you formally withdrew your consent may continue to be used and disclosed by the investigators for the purposes described above.

To formally withdraw your consent for participation in this research study you should provide a written and dated notice of this decision to the principal investigator of this research study at the

address listed on the first page of this form. Any identifiable research or information recorded for, or resulting from, your participation in this research study prior to the date withdrawn from participation may continue to be used and disclosed by the investigators for the purposes described.

Your decision to withdraw your consent for participation in this research study will have no effect on your current or future relationship with the University of Pittsburgh. Your decision to withdraw your consent for participation in this research study will have no effect on your current or future medical care at a UPMC hospital or affiliated health care provider or your current or future relationship with a health care insurance provider.

At the time of withdrawal we ask that all study equipment (i.e., blood pressure cuff and monitor, heart rate variability monitor, paper diary, activity monitors) be returned to the study team.

If I agree to participate in this research study, can I be removed from the study without my consent?
It is possible that you may be withdrawn from the study by the researchers, for example, due to not following the instructions provided to you by the investigators and staff, or if continued participation is determined to be not medically in your best interest (for example if your blood pressure exceeds 180/110).

VOLUNTARY CONSENT

The above information has been explained to me and all of my current questions have been answered. I understand that I am encouraged to ask questions, voice concerns or complaints about any aspect of this research study during the course of this study, and that such future questions, concerns or complaints will be answered by a qualified individual or by the investigator(s) listed on the first page of this consent document at the telephone number(s) given. I understand that I may always request that my questions, concerns or complaints be addressed by a listed investigator.

I understand that I may contact the Human Subjects Protection Advocate of the IRB Office, University of Pittsburgh (1-866-212-2668) to discuss problems, concerns, and questions; obtain information; offer input; or discuss situations in the event that the research team is unavailable.

By signing this form I agree to participate in this research study. A copy of this consent form will be given to me.

____
Participant's Printed Name

____________________________________ ____________________
Participant's Signature Date

CERTIFICATION OF INFORMED CONSENT:

I certify that I have explained the nature and purpose of this research study to the above-named individual, and I have discussed the potential benefits and possible risks of study participation. Any questions the individual has about this study have been answered, and we will always be available to address future questions, concerns or complaints as they arise. I further certify that no research component of this protocol was begun until after this consent form was signed.

____________________________________ ____________________________________
Printed Name of Person Obtaining Consent Role in Research Study

____________________________________ ____________________________________
Signature of Person Obtaining Consent Date
